# Structurally Ordered NIPUs via Catalyst-Free Synthesis with Hard Segments Based on Erythritol and a Long-Chain Diamine

**DOI:** 10.3390/molecules30142912

**Published:** 2025-07-10

**Authors:** Edyta Hebda, Karolina Wróbel, Aleksandra Cieślik, Kinga Szołdrowska, Jan Ozimek, Paulina Zając, Konstantinos N. Raftopoulos, Krzysztof Pielichowski

**Affiliations:** Department of Chemistry and Technology of Polymers, Cracow University of Technology, Warszawska 24, 31-155 Krakow, Poland; karolinajuliawrobel@gmail.com (K.W.); ola.cie1999@gmail.com (A.C.); kinga_szoldrowska@poczta.fm (K.S.); jan.ozimek@pk.edu.pl (J.O.); paulina.zajac@pk.edu.pl (P.Z.); konstantinos.raftopoulos@pk.edu.pl (K.N.R.); krzysztof.pielichowski@pk.edu.pl (K.P.)

**Keywords:** dicarbonate erythritol, polyhydroxyurethane, segmented non-isocyanate polyurethane, structure, phase separation

## Abstract

A series of linear isocyanate-free polyurethanes (NIPUs) were obtained via the aminolysis of erythritol dicarbonate (EDC) with polyethers (diamino-PEG, diamino-PPO, and diamino-PEG/PPO) and 1,12-diaminododecane (DADD), which acts as a chain extender to form hard segments. The obtained NIPUs contained different concentrations of DADD relative to the polyether (72.5–80 wt%). A detailed chemical structure analysis of the synthesized NIPU was performed using a combination of FTIR and ^1^H NMR. FTIR spectra confirmed that the EDC/DADD segments formed a network of hydrogen bonds. This is reflected in WAXD diffractograms showing ordered crystalline domains originating in DADD. The reflections assigned to the EDC/DADD segments exhibited changes in their position and intensity with decreasing concentration, indicating an increase in interplanar spacing and a loss of higher-order order. WAXD also showed that the soft segments of PEG and PEG/PPO retain their ordered crystal structure regardless of the EDC/DADD content. At a larger length scale, SAXS revealed similar micromorphology for the different polyethers, with a broad peak indicating long-range order in the EDC/DADD-rich segments and a weak separation of the soft and hard phases. DSC analyses confirmed the complex phase behavior, where the PEG-based materials showed melting of crystalline fragments, and the amorphous PPO showed a glass transition. DMA indicated the stability of the glass transition temperature in the PPO samples and the presence of an unusual structural transition. The results emphasize the influence of the type of poly(ether) on the thermal and microphase properties of the studied non-isocyanate polyurethanes.

## 1. Introduction

Polyurethanes are an important class of polymers, widely used due to their versatility; they come in various forms, such as foams, elastomers, adhesives, sealants, or coatings, which allows for their broad use in many industries, including the biomedical, automotive, and construction sectors [1,2]. The vast possibilities of modifying polyurethanes’ properties by selecting appropriate raw materials with different structures and functionalities make it possible to design macromolecular materials for various applications [3]. Conventional polyurethanes are produced according to the method developed by Bayer before the outbreak of World War II, from polyols and isocyanates [4]. However, one of the main challenges related to the safety and synthesis of these materials is the toxic nature of isocyanates. The harmful effects of isocyanates, as well as their precursor—phosgene—on human health and the environment, have led to the introduction of regulations restricting their use. Therefore, the search for alternatives that would be safer and more environmentally friendly has become one of the main directions of development of the polyurethane industry [5,6].

A viable option is the synthesis of isocyanate-free polyurethanes (NIPUs), which do not require the use of toxic diisocyanates [7,8].

Moreover, replacing isocyanates in the synthesis of NIPU makes the reaction mixture less sensitive to moisture, unlike traditional polyurethanes, where the reaction of isocyanates with water leads to the formation of amine groups and carbon dioxide [9]. On the downside, isocyanate-free polyurethanes are characterized by a longer polymerization time, which makes their production a slower and more time-consuming process [8].

Currently, one of the most promising methods to synthesize isocyanate-free polyurethanes (NIPUs) is the aminolysis of cyclic carbonates [10,11,12]. This approach closely resembles the conventional method of polyurethane synthesis [13] while avoiding the use of harmful isocyanates at all stages [8,14]. Cyclic carbonates are nontoxic and moisture-insensitive, eliminating the need for special storage conditions [15]. Moreover, the aminolysis process does not produce volatile organic compounds or byproducts requiring separation. The resulting polyhydroxyurethanes (PHUs) are characterized by the simultaneous formation of urethane and hydroxyl groups, primarily secondary hydroxyls, upon the ring-opening of cyclic carbonates [16]. The ratio of primary to secondary hydroxyl groups depends on the solvent and the chemical structure of both amines and cyclic carbonates [16,17,18].

The presence of hydroxyl groups adjacent to urethane bonds significantly affects the thermomechanical properties of PHUs [19,20,21], often distinguishing them from conventional polyurethanes [22,23]. Specifically, hydroxyl groups disrupt hydrogen bonding within rigid segments and enhance compatibility between rigid and flexible segments, especially in polyether-based PHUs. This increased miscibility, while improving homogeneity, can suppress microphase separation—a critical factor in tuning material performance—and thereby diminish mechanical strength and thermal stability [19].

Thus, the composition and structural nature of the rigid and flexible segments play a pivotal role in determining whether phase separation and ordered microstructures can be achieved in NIPUs. The flexible segments influence chain mobility and interphase interactions, while the rigidity and polarity of hard segments, and their ability to form hydrogen bonds, determine the extent of phase segregation and internal cohesion.

Hydroxyl groups also contribute to the formation of intra- and inter-molecular hydrogen bonds, increasing the chemical resistance of PHUs compared to conventional PUs [8,24,25]. However, these same groups hinder crystallization, rendering PHUs predominantly amorphous or only semi-crystalline with limited capacity for microphase separation [8,23,26,27]. Additionally, they increase water absorption, leading to material plasticization and reduced stiffness [24].

Recent studies by Torkelson and collaborators [19,28,29] underscore the critical influence of segmental architecture on the final properties of segmented PHUs. In these hybrid materials, the use of flexible polyether segments such as polyethylene oxide (PEO) facilitated strong hydrogen bonding between ether oxygen atoms in the soft segments and hydroxyls in the hard segments; this resulted in significant phase mixing. Conversely, the introduction of flexible segments like polypropylene glycol (PPG), with bulkier structures and less accessible ether oxygens, hinders hydrogen bond formation, thus promoting phase separation. PHUs containing polytetramethylene oxide (PTMO) segments demonstrated an intermediate behavior, showing partial suppression of hydrogen bonding and enabling controlled microphase organization [29]. These findings clearly indicate that precise control over both the type and the ratio of soft and hard segments is essential for tuning the morphology and performance of PHUs [21].

The low reactivity of many multifunctional carbonates—such as those derived from biopolyols, epoxidized terpenes, or vegetable oils—necessitates the use of catalysts like triethylamine, DABCO, DBU, TBD, and thioureas in PHU synthesis [13,30,31]. The purpose of their use is to increase the rate of reaction and enable processes to be carried out under milder conditions. Among these, TBD stands out due to its high catalytic efficiency [32,33]. Nevertheless, even with catalysts, the solution polyaddition method requires elevated temperatures and prolonged reaction times [34,35,36,37,38,39,40]. As noted by Besse et al. [20,41,42,43,44], the extended polymerization time can promote side reactions, making precise stoichiometric control difficult and thereby limiting the achievable molar mass of the final polymer. Moreover, the presence of a catalyst may, in some cases, lead to undesirable effects, such as degradation or a change in the product microstructure.

A sustainable route for synthesizing five-membered dicarbonates involves the carbonation of sugar-derived tetraols. A key example is erythritol dicarbonate (EDC), derived from meso-erythritol, which is optically inactive and benefits from an inductive effect that enhances its reactivity relative to other carbonates [45,46]. Due to this increased reactivity, the synthesis of NIPU with the participation of EDC can be carried out without a catalyst, which simplifies the process and reduces the risk of undesirable side reactions. Studies of EDC aminolysis in DMSO at room temperature reveal that the reaction rate and regioselectivity are heavily influenced by stereochemistry. The resulting hydroxyurethanes predominantly contain secondary hydroxyl groups (about 85%) [45], further emphasizing the importance of segmental composition and functionality in determining hydrogen bonding patterns, phase behavior, and, thus, various material properties. In addition, Le Goupil [46] showed that when EDC is combined through aminolysis with 1,12-diaminododecane (DADD), the resulting material exhibits crystallinity and, thus, resembles the behavior of hard segments, as understood in the context of conventional polyurethanes. In this work, we expand this concept by preparing and studying segmental systems consisting of the EDC/DADD homopolymer with flexible polyether chains. The aim is to produce phase-separated materials and study the effects of composition and the nature of the polyethers on phase morphology and thermomechanical properties. The phase separation behavior of EDC-derived NIPU segmented copolymers with polyethers and DADD has not been investigated or reported yet. The present study shows that the choice of a rigid segment can significantly tune the phase separation state in segmented PHUs.

More specifically, we synthesized NIPU using erythritol dicarbonate, three kinds of polyether (PEG, PPO, or PPO/PEG), and 1,12-diaminododecane (DADD) in different ratios of flexible segment (i.e., polyether) to chain extender (1,12-diaminododecane). The selection of these three polyethers was made to investigate the influence of the structure and properties of soft segments on phase separation. PEG (polyethylene glycol) is a linear, highly hydrophilic polyether, while PPO (polypropylene glycol) is hydrophobic and amorphous. The third polyether, designated as PPO/PEG, is actually a triblock copolymer from the poloxamer family, containing PPO blocks in the core and PEG at the chain ends. It was chosen due to its amphiphilic nature and potential self-assembly ability, which makes it an interesting material for studying the influence of soft segment architecture on copolymer morphology. The use of 1,12-diaminododecane (DADD) was motivated by its well-defined, aliphatic structure, which can promote the ordering of hydroxyurethane (rich hard segments (EDC/DADD sequences) and, thus, enhance phase separation.

**Scheme 1:**
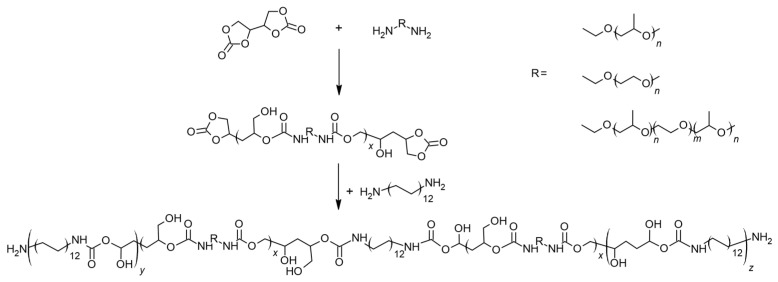
Reaction schemes for the synthesis of PEG-, PPO-, and PEG/PPO-based PHUs with 1,12-diaminododecane.

Linear polyhydroxyurethanes were obtained in the reaction of difunctional cyclic erythritol dicarbonate with amine components, including polyethers (diamino-PEG, diamino-PPO, or diamino-PPO/PEG) and a shorter amine, 1,12-diaminododecane. The details of the synthesis are given in Section 4 and presented in Scheme 1.

Erythritol dicarbonate (EDC) reacts with a mixture of two amines in equimolar amounts to EDC dicarbonate (1:1). In the first step, EDC reacts with the selected polyether, and then in the second step, 1,12-diaminododecane (DADD) is added to the system to form a linear system. The polyether to DADD mass fractions are shown in Table 1, and the molar ratio of EDC to total amine (poly-ether and DADD) was 1:1. In the following, the samples will be denoted as EDC-<polyether>-<mass fraction of the polyether in the amine component in wt%>, e.g., EDC-PEG-27.5 denotes a sample with 27.5 wt% PEG and 72.5 wt% DADD in the amine component.

All polyethers used in the course of this study had a molecular weight of approximately 2000 Da, whereas Jeffamine@ED2003 is characterized by a triblock structure with 39 repeating units in the central m block and a total of 6 repeating units in the n and l blocks, resulting in a predominantly PEG-based backbone. The introduction of the selected polyether with a higher molecular weight to the reaction resulted in the possibility to complete the reaction before the introduction of the shorter DADD acting as a chain extender. It should be emphasized that no catalyst was used in the synthesis of NIPU, as the reactivity of erythritol dicarbonate is sufficient for a successful synthesis.

## 2. Results and Discussion

### 2.1. FT-IR

**Figure 1:**
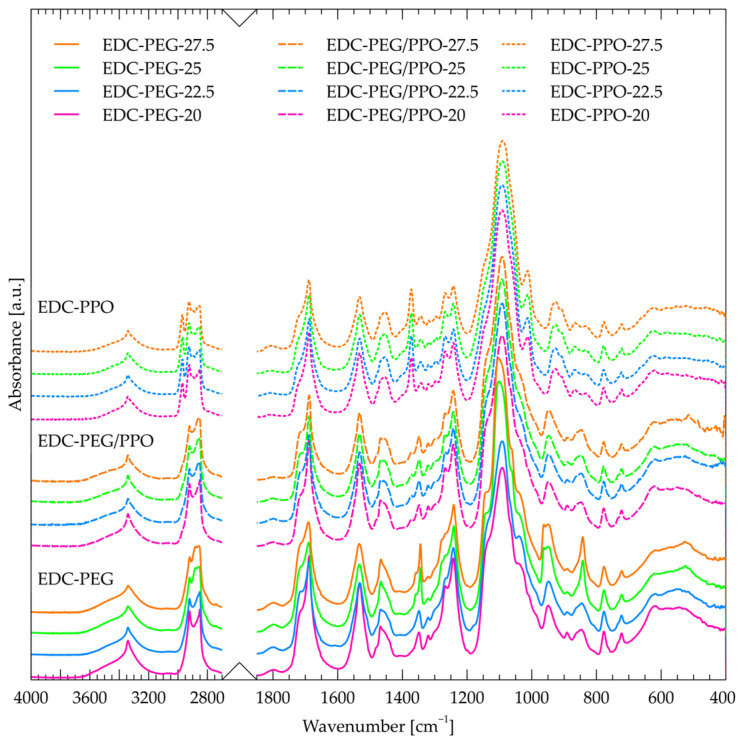
ATR-FTIR spectra of non-isocyanate polyurethanes synthesized using PEG and PPO substrates.

Figure 1 shows the ATR-FTIR spectra of all obtained materials. The band corresponding to the residual cyclic carbonate (ca. 1800 cm^−1^ [19]) shows negligible intensity, which indicates a high degree of conversion and a high molar mass exceeding the entanglement limit. The obtained spectra qualitatively correspond to those reported for systems synthesized in a similar way [47].

The bands at 3300 and 3315 cm^−1^ are assigned to the hydrogen bond stretching vibrations of OH and NH groups, respectively. Mishra et al. [48] described three types of NH stretching vibrations in segmented polyurethanes at 3499, 3352, and 3306 cm^−1^. They are assigned to the following:

- Free NH groups;
- NH groups that are connected to carbonyl in hard segments—type I;
- NH groups that are connected to ether oxygen in soft segments—type II.

**Figure 2:**
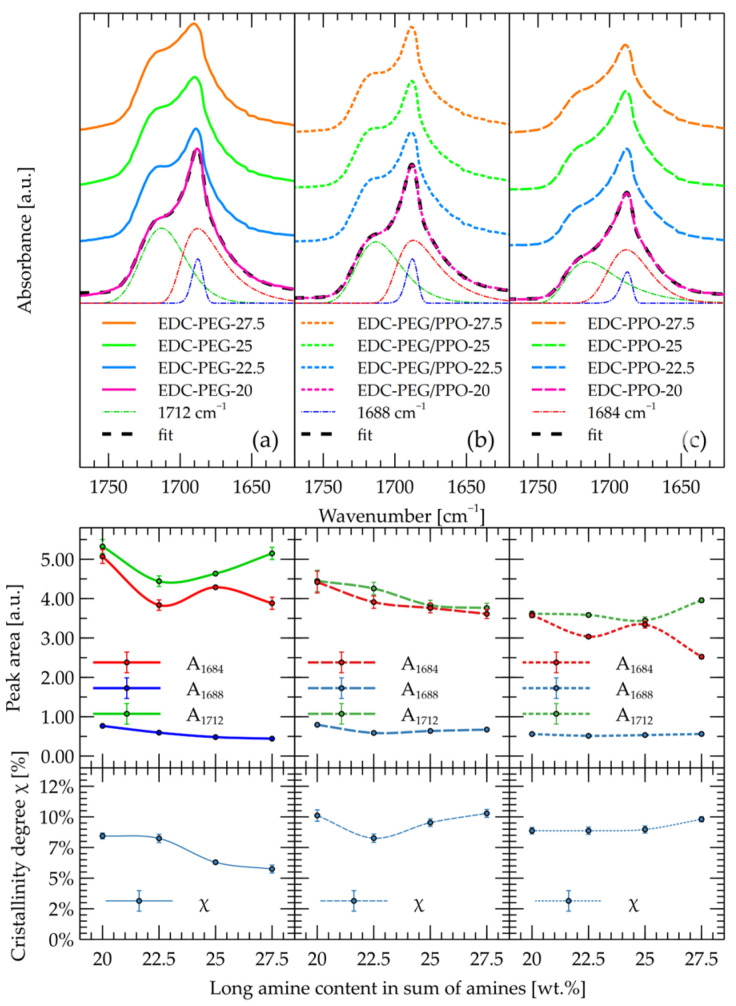
ATR-FTIR spectra of all materials in the amide I region related to the carbonyl of the urethane group. (**a**) EDC-PEG; (**b**) EDC-PEG/PPO; (**c**) EDC-PPO. The second row shows the intensities of the bands, as determined by the deconvolution. The third row shows the ratio χ, associated with phase separation.

**Scheme 2:**
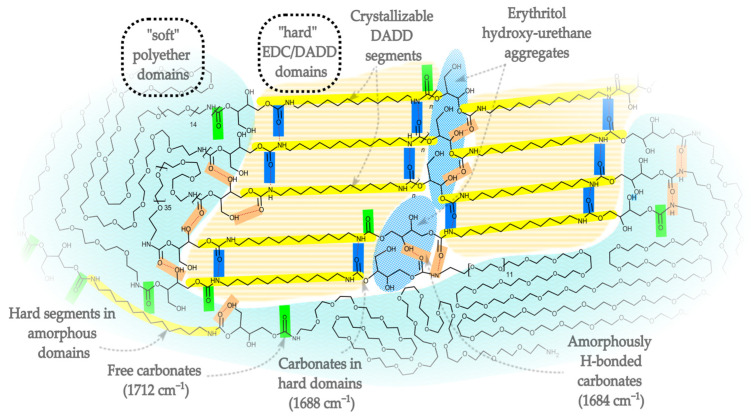
Simplified illustration of phase-separated NIPU structure showing polyether hydrophilic domains, crystallized DADD segments, erythritol hydrophilic domains, and hydrogen-bonded vs. free carbonate domains (IR peaks: 1712 cm^−1^, 1688 cm^−1^, and 1684 cm^−1^).

In our work, all obtained NIPUs showed a sharp band at about 3340 cm^−1^, which suggests that NH groups participate in type I hydrogen bonding. The second region that was considered is the location of absorption bands at 1680–1720 cm^−1^ originating from the formation of carbonyls in polyurethanes. Various studies explore the influence of hydrogen bonding on the C=O resonance frequencies in phase-separated PU systems [21,49], and the carbonyl band often consists of two components: free C=O (not hydrogen-bonded): ~1720 cm^−1^ and hydrogen-bonded C=O: ~1680–1700 cm^−1^. The ratio of the band area of bound to free carbonyls can be used as an indicator of the degree of hard segment ordering—and thus the degree of phase separation. Shifts in this band indicate the strength and number of hydrogen bonds (stronger bonds cause a shift toward lower wave numbers). The region associated with the stretching vibrations of urethane carbonyls (1680–1700 cm^−1^) appears to consist of two strongly overlapping components, roughly centered around 1712 cm^−1^ and 1684 cm^−1^ (Figure 2). These two signals are routinely observed in polyurethanes [50], as well as in polyamides [51]. The component with the highest wavenumber—1712 cm^−1^—is assigned to carbonyls without hydrogen bonds. The component at a lower wavenumber—1684 cm^−1^—corresponds to the occurrence of carbonyls that are hydrogen-bonded in a disordered manner. The deconvolution of this region (Figure 2) was performed using the functions proposed by Stancik [52], which describe macromolecules well. The proposed model shows that there is another component in the form of a rather sharp band at about 1688 cm^−1^ with the lowest intensity. We can attribute it to the presence of hard segments that create ordered crystal domains. Such an approach was used previously in classical polyurethanes [53]. Scheme 2 shows a simplified illustration of the microphase-separated structure of the NIPU system, indicating the spatial distribution of various hydrogen-bonded and free carbonyl domains. This diagram shows the correspondence between the hard and soft segment distribution and the type of hydrogen bonds observed in the FTIR spectra. A distinction is visible between free carbonyls (1712 cm^−1^), randomly hydrogen-bonded carbonyls (1684 cm^−1^), and those in ordered crystalline segments (1688 cm^−1^). The presence of these segments correlates with the occurrence of the band at 3340 cm^−1^, assigned to NH groups that are connected to carbonyl in the hard segments.

It is observed that the change in the polyether:DADD ratio does not cause significant changes in the surface area of this component (at 1688 cm^−1^) (Figure 2b). This may be due to subtle changes in the mass ratios. The change in the PPO:DADD ratio does not cause significant changes in the components at 1712 and 1684 cm^−1^, while in the case of the change of the PEG:DADD and PEG/PPO:DADD ratios, a decrease in the surface area of both components is observed first, and for the highest ratios, an increase in non-hydrogen-bonded carbonyls occurred.

### 2.2. ^1^H NMR

**Figure 3:**
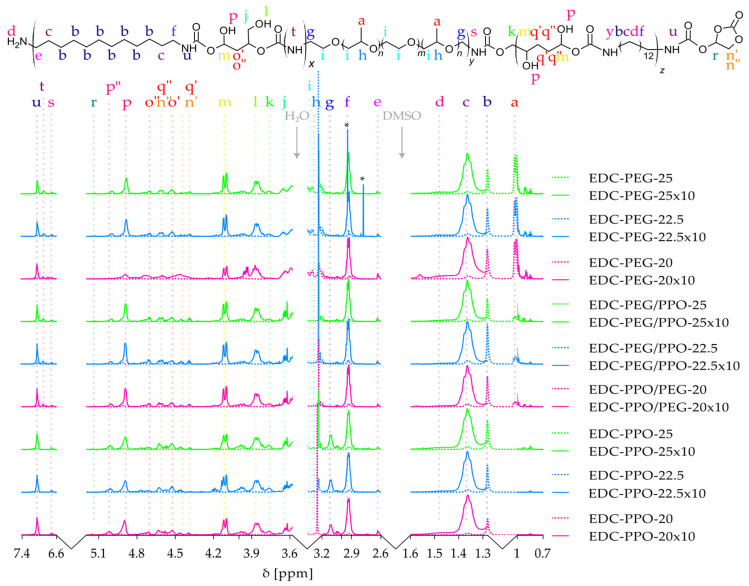
^1^H NMR spectra (DMSO-d_6_, 400 MHz) of all materials. Each spectrum is overlaid twice: (1) a dotted line at true intensity (used for integration), and (2) a solid line, amplified 10× to reveal weak signals such as NH and OH resonances. Letters (a–t) correspond to proton environments labeled in the chemical structure above, with the dashed vertical lines aiding in peak identification. Key features include methylene and methine resonances (*), NH and OH groups (p^H^: hydrogen-bonded and p: unbonded), and weak signals from unreacted terminal groups.

^1^H NMR was performed to confirm the chemical structure of the obtained polymers and to elucidate the detailed structural features of the polymer backbone and the end groups. The spectra of each material are combined in Figure 3. Spectra were recorded in DMSO-d_6_ to preserve and resolve exchangeable NH and OH protons. Various polymer formulations—based on biscyclic erythritol dicarbonate (EDC) and different diamines (DADD, PEG-diamine, PPO/PEG-based copolymer, and PPO-diamine)—were analyzed.

To enhance readability across a broad dynamic range of signal intensities, each spectrum was plotted twice: the true-intensity trace is shown as a dotted line (used for integration), and a 10-fold amplified overlay is shown as a solid line, allowing for the detection of low-intensity signals such as NH and OH protons [46,53]. This dual plotting reveals subtle features that would otherwise be masked by dominant methylene signals.

Each proton environment is labeled with lowercase letters (a–t) in the chemical structure above the spectra and assigned to corresponding signals using dashed vertical guide lines. The signal assignments confirm the expected PHU structure and allow for the differentiation of backbone units, end groups, and regioisomeric ring-opening products. Notably, we see the following:

- Methyl groups from PPO (a) appear at δ 0.85–0.95 ppm as a doublet, broadened in mixed PEG-PPO systems.
- Internal aliphatic CH_2_ groups (b) from DADD are seen as broad multiplets at δ 1.2–1.5 ppm, while β-methylene groups (c) are slightly downfield (δ ~1.6–2.0 ppm) due to proximity to urethane and ether linkages.
- Terminal NH_2_ and adjacent CH_2_ signals (d, e) are visible in some spectra as low-intensity features, indicating slight chain-end functionality.
- Methylene groups adjacent to NH (f, g) in DADD and PEO segments appear at δ ~2.9–3.1 ppm.
- PPO methine and ether CH_2_ (h, i) resonate at δ ~3.3–3.4 ppm and δ ~3.5–3.7 ppm, respectively, forming part of the PEO/PPO backbone.
- CHOH-associated CH_2_ and CH protons (j, m, o) appear from δ 4.0 to 4.7 ppm, with signal complexity reflecting regioisomeric ring opening.
- Secondary OH groups (p) show broad signals at δ 4.8–5.0 ppm, and a distinct multiplet at δ ~5.02 ppm (pH) is attributed to hydrogen-bonded CH–OH. The peak pH may be due to OH ^…^ O=C—the hydroxyl groups, bonded with the urethane group, and is responsible for dividing the FTIR band for urethanes into the 1684 cm^−1^ band.
- Notably, the CHOH–OH signal splitting varies with diamine structure: a triplet is observed when PPO is used (due to asymmetric, rigid environments near the urethane), while a doublet appears in PEG-containing systems where greater flexibility and symmetry may reduce additional coupling.

The presence of urethane NH groups (r, s, t) is confirmed by broad downfield signals at δ ~6.8–7.5 ppm, with minor chemical shift differences depending on whether DADD-, PEG-, or PPO-derived urethane segments are present.

This spectral analysis, particularly the observation of CHOH and OH signals in expected regions with the correct multiplicity, provides strong confirmation of the successful ring-opening addition of amines to cyclic carbonates, resulting in a linear PHU with pendant primary and secondary hydroxyl groups.

### 2.3. WAXD

**Figure 4:**
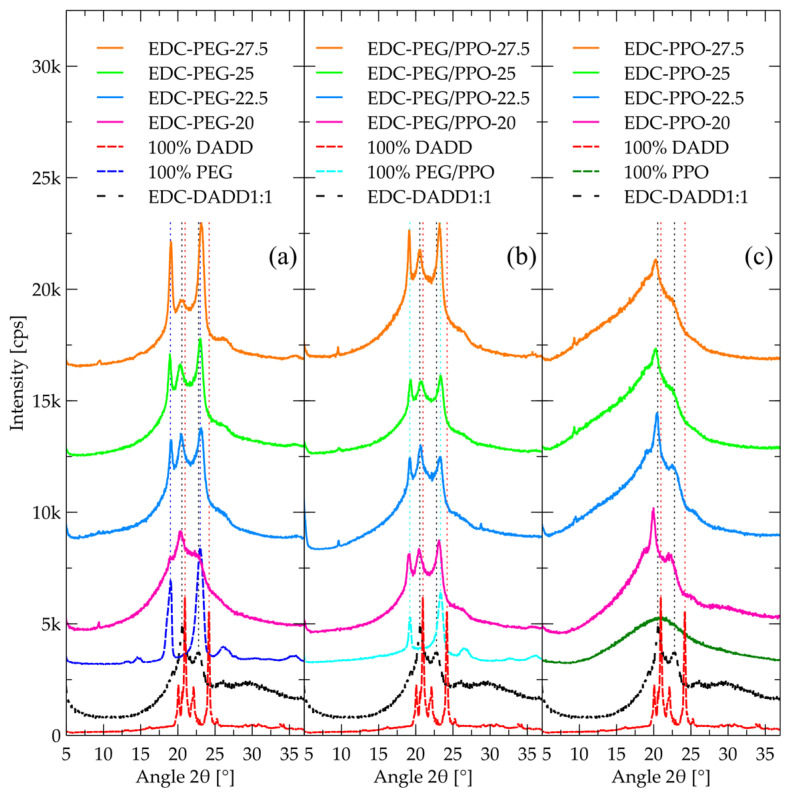
WAXD patterns of reagents and PHU based in (**a**) PEG, (**b**) PEG/PPO copolymers and (**c**) PPO with different content in relation to DADD.

X-ray diffraction analysis (WAXD) was performed to investigate the effect of 1,12-diaminododecane (DADD) content on the structure of isocyanate-free polyurethanes (NIPU) (Figure 4).

DADD, as a linear aliphatic diamine, is incorporated into the hard segments and significantly affects their organization. The WAXD results showed that the diffraction reflections from PEG soft segments and from PEG/PPO (~19° and ~23° 2θ) [54] remain unchanged regardless of the amount of DADD introduced. This means that PEG and PEG/PPO maintain their ordered, crystalline structure and are not disturbed by the presence of hard segments. In the case of PPO-based segments, which are typically amorphous, no distinct reflections were observed, which confirms their non-crystalline nature. In contrast, the reflections associated with DADD segments change significantly with the increase in their fraction in the material. The main diffraction peak is shifted towards lower values of the 2θ angle, which indicates an increase in the interfacial distance and suggests a more loosely packed arrangement of these segments. At the same time, the second diffraction reflection (observed in pure DADD) disappears, which may indicate the loss of higher-order order. The lack of changes in the position of the PEG and PEG/PPO reflections, with the simultaneous shift and disappearance of the signals associated with DADD, confirms that the changes in the structure concern only the hard segments. In the NIPU spectra based on PEG and PEG/PPO, a reflection appears at an angle of ~20°, and for PPO (~20° and 22.7°) originating from EDC/DADD segments, the intensity decreases with the increase in the soft segments. These observations lead to the conclusion that DADD, along with EDC, forms rigid, partially ordered domains, the spatial organization of which decreases with the increased content of this diamine, which is consistent with the mechanism of the microphase separation of hard and soft segments in NIPU. The obtained results are consistent with previous reports on the behavior of polyurethanes based on PEG and aliphatic segments. It has been emphasized in the literature that PEG forms stable crystalline domains resistant to the influence of hard segment modifications, and long aliphatic diamines, such as DADD, can form partially ordered structures, the order of which depends on the conditions of synthesis and concentration [19,55].

### 2.4. SAXS

**Figure 5:**
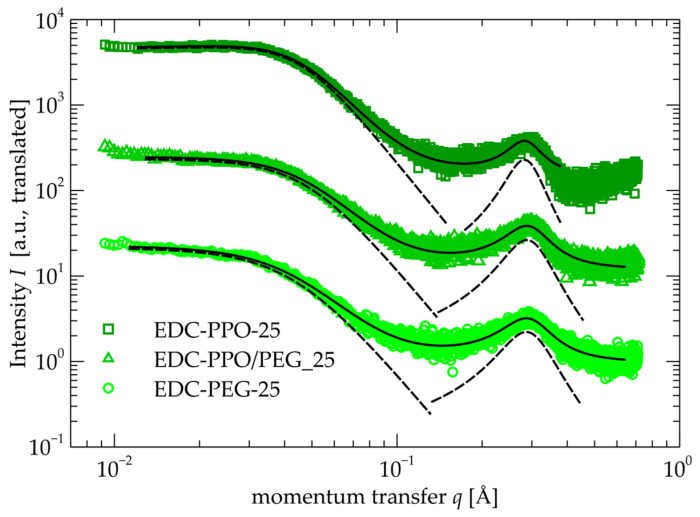
SAXS curves recorded with the samples, including 25% of each of the polyethers. Results of the fit, along with the components of the model, are also included. Curves have been translated for clarity.

The SAXS curves (Figure 5) are very similar to each other, indicating very similar micromorphology between the three polyethers. This is somewhat unexpected when taking into account that samples containing PEG chains contain crystalline structures, as shown by DSC (Section 2.5) and WAXD (Section 2.3). Such regions, however, do not give rise to new features in the length scale accessible by the experiment. This should be attributed to a small contrast between amorphous and crystalline domains when compared to that between EDC/DADD and polyether segments.

The curves have two main features. On the low *q* side, there is a prominent drop in the signal that starts at the 0.04–0.05 Å^−1^ region. For the PPO-based sample, a very weak upturn is observed before the drop. This should be attributed to a weak phase separation between EDC/DADD-rich regions and polyether-rich regions. It is worth noting that in conventional segmental polyurethanes, well-defined peaks related to microphase separation occur in this q range [56,57,58,59,60,61].

At 0.3 Å^−1^, a relatively broad peak is observed for all materials under investigation. This is indicative of strong ordering with periodicity in the order of a few nm at a length scale much smaller than that of the microphase separation, i.e., within one of the domains. Its location is seemingly the same for all materials, which points to its attribution to ordering within EDC/DADD domains, presumably the distance between urethane units, possibly coordinated with each other with hydrogen bonds.

In order to draw more quantitative results, we fitted model functions to the curves. The drop on the low *q* side was modelled adequately with a Teubner–Strey function (1), which is typically used for weakly phase-separated systems [62,63,64].

(1) $$I\left(q\right)=\frac{A}{a_{2}+c_{1}q^{2}+c_{2}q^{4}}$$

where $a_{2}={\left(1+{\left(\frac{2\pi \xi_{TS}}{d_{TS}}\right)}^{2}\right)}^{2}$, $c_{1}=-2\xi_{TS}^{2}{\left(\frac{2\pi \xi_{TS}}{d_{TS}}\right)}^{2}+2\xi_{TS}^{2}$, and $c_{2}=\xi_{TS}^{4}$. In this model, A is a scale parameter, $\xi_{TS}$ is a correlation length, and $d_{TS}$ is a measure of the periodicity of the phase separation related to the domain size.

The peak around 0.3 Å^−1^ was modelled with a Lorentz peak function (2) [65].

(2) $$I_{L}\left(q\right)=\frac{B}{1+{\left(\xi_{L}\left(q-q_{0}\right)\right)}^{2}}$$

where B is a scale parameter, $\xi_{L}$ is a correlation length, and $q_{0}=\frac{2\pi }{d_{L}}$, with $d_{L}$ being the characteristic distance between scatterers. As a background, a constant term was used, along with a power law, to account for an upturn at high q values.

The results of the fitting are collected in Table 2.

The d*_L_* values (periodicity) of the low length scale order (at this point, attributed to the ordering of the hydroxyurethane segments in EDC/DADD segments) confirm the observation that it is the same for all three samples. The correlation length parameter of the Lorentz peak is also similar between the samples and somewhat smaller than the periodicity, indicating that the order is very localized, i.e., does not extend beyond a few units.

Turning our attention to the feature at low *q* values, here, the correlation length parameter *ξ_TS_* is also smaller than the repeat distance, indicating a very weak and not-well-periodically phased separation. The length scale of the phase separation *d_TS_* shows very little variation with the type of polyether. However, it seems that the PPO-based material forms smaller structures than the PEG-based versions, and their copolymer forms structures of intermediate periodicity. The reason for this observation is not clear at this point, but it should be sought in the complex correlation between the molar mass of the polyethers, the packing of their segments, and the amount of hard segments diluted in the soft phase. The ratio of correlation length to periodicity parameters is higher for the PPO-based material, possibly indicating better microphase separation, with higher incompatibility between the PPO and EDC/DADD segments when compared to PEG. This is alsocompatible with the fact that the upturn before the drop of the signal is more visible for the PPO-based material.

### 2.5. DSC

**Figure 6:**
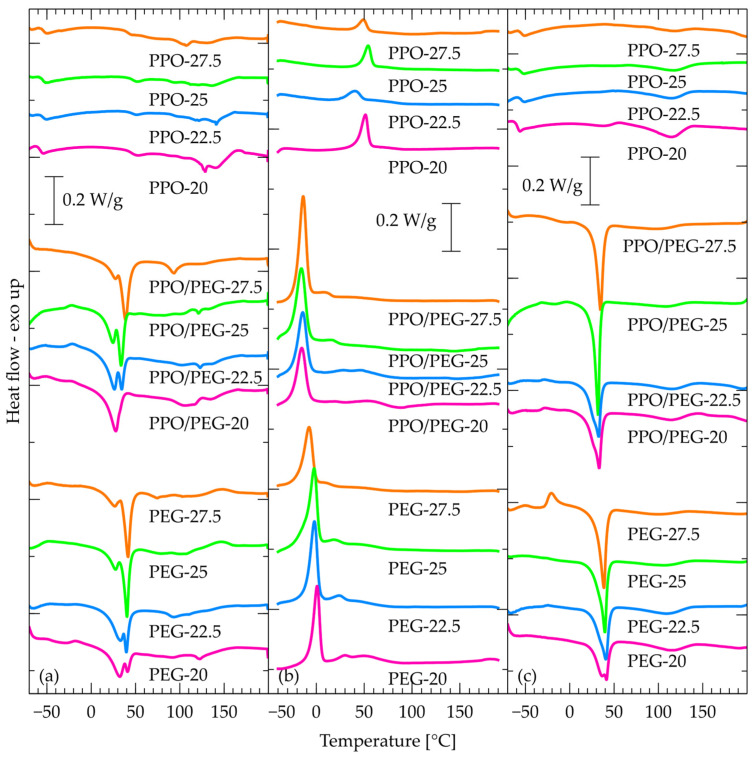
DSC curves recorded for all materials under investigation. (**a**) First heating run; (**b**) cooling run; (**c**) second heating run. The prefix EDC in the code name of the samples has been omitted for clarity.

The DSC profiles (Figure 6) indicate that the materials have quite a complex phase behavior.

Starting from the first heating run (Figure 6a), the PEG- and PPO/PEG-based materials show a strong, sharp, and complex endothermic peak around 40 °C, corresponding to the melting of crystalline regions originating from the relatively long PEG segments [66]. The fact that PEG chains can form strong crystalline structures, despite having a relatively small molar mass (being the minority component and being copolymerized with other types of segments (EDC/DADD ones and PPO fragments)), shows that there is at least some phase separation, leading to relatively “pure” PEO regions. In any case, this crystallinity likely provides structural integrity at room temperature, as will be confirmed by DMA (Section 2.6). The fact that the melting happens close to human body temperature may be of interest for the design of smart materials for biomedical applications. The melting of PEG chains is followed at higher temperatures by a series of thermal events, presumably corresponding to the gradual disordering of the ordered structures observed by SAXS (Section 2.4) and WAXD (Section 2.3). We will return to this point later when discussing the second heating run. This disordering is better observed for the PPO-based materials; however, no sharp melting endotherm is observed due to the amorphous nature of PPO. Instead, a glass transition step is observed around −60 °C. This should be attributed to the amorphous PPO segments. The fact that it is observed at a somewhat higher temperature than that of the pure polyether (around −70 °C [67]) indicates that it is mixed to some extent with the more rigid EDC/DADD segments. The fact that its temperature or intensity does not show a dependence on composition indicates that, in this composition range, the amount of diluted EDC/DADD segments in the soft phase is constant; in other words, the soft phase is saturated with hard segments, and additional ones form the hard microdomains.

Proceeding to the cooling curves of the PPO-based materials (Figure 6b), well-defined single exothermic peaks emerge in the range of 20–60 °C. Given that PPO is an amorphous polymer, this peak should be attributed to the ordering of EDC/DADD segments, as argued in the SAXS and WAXD results sections. This is another strong indication of the weak yet existent thermoplasticity of the materials. Similar peaks also appear in the same region for the materials based on the other two polyethers; however, these peaks are double, much broader, and occur at slightly lower temperatures (higher supercoolings). This correlates well with the indications from SAXS that phase separation is stronger for the PPO-based materials due to higher incompatibility between the hydrophilic hydroxyurethane-rich segments and the hydrophobic PPO.

It is also interesting to note that the intensity, temperature, and width of the ordering exotherms do not follow well-defined trends with the fraction of the polyethers in the materials. This indicates a complex dependence of ordering dynamics on the composition. At this point, we would like to refrain from speculations regarding the nature of ordering and, thus, the mechanisms driving it. In order to provide more concrete discussion on the matter, a combination of static and dynamic DSC- and temperature-resolved SAXS experiments should be conducted following the methodology used in the seminal works of Koberstein in the 1980s–1990s for conventional polyurethanes [56,57,68,69,70,71,72].

PEG and PPO/PEG samples exhibit yet another, much stronger and sharper ordering endotherm in the region of 0 to −20 °C. This should be attributed to the crystallization of the relatively long PEG units. The crystallization needs some more supercooling when compared to the pristine polyether [65]. This peak also does not show well-defined trends with composition. The mechanism behind this observation is also not trivial to understand, as it is intimately connected to the microphase separation development and simultaneous emergence of polyether-rich regions [73].

We finally turn our attention to the second heating curves (Figure 6c). The PEG melting is now sharper, more intense, and occurs at somewhat higher temperatures when compared to the first heating run. This indicates a more homogeneous crystallization when the materials are cooled down in a controlled manner, presumably as a result of the time given to the material for equilibration. The series of weak thermal events at higher temperatures is substituted by a single, shallow, and broad endothermic peak around 130–140 °C. This should be attributed to the disordering of the EDC/DADD-rich domains, which had formed order during cooling. Two indications for this attribution of the peak are that it occurs at temperatures well above the ordering and that its location is sensitive neither to composition nor to the kind of polyether in the system. In addition, although quantification is challenging due to the width of the peaks, we can state that for materials with low polyether-high DADD content, the peaks are better discerned, and this is another strong indication that they originate from the EDC/DADD-rich domains. In any case, it can be stated that the materials are thermoplastic. In addition, the temperature at which this reversible process occurs allows for the easy, low-temperature reprocessing of the materials, enabling their easy recycling. This renders them potentially useful as hot adhesives.

On the low-temperature side, for the PPO-based materials, the glass transition step, also observed during the first heating, is visible around −60 °C.

The PEG-containing materials are strongly crystalline, as we have already observed. Hence, no well-discernible glass transition steps are visible. This should be related either to the small amount of amorphous polyether segments reducing the intensity of the phenomenon or to extreme broadening of the effect due to the heterogeneity. Nevertheless, for one material (EDC-PEG_27.5), which also exhibited cold crystallization, a step is visible around −45 °C. The glass transition temperature of PEG is known to vary strongly with crystallinity, and therefore, we would like to refrain from any comments regarding this particular value [67,74].

### 2.6. DMA

**Figure 7:**
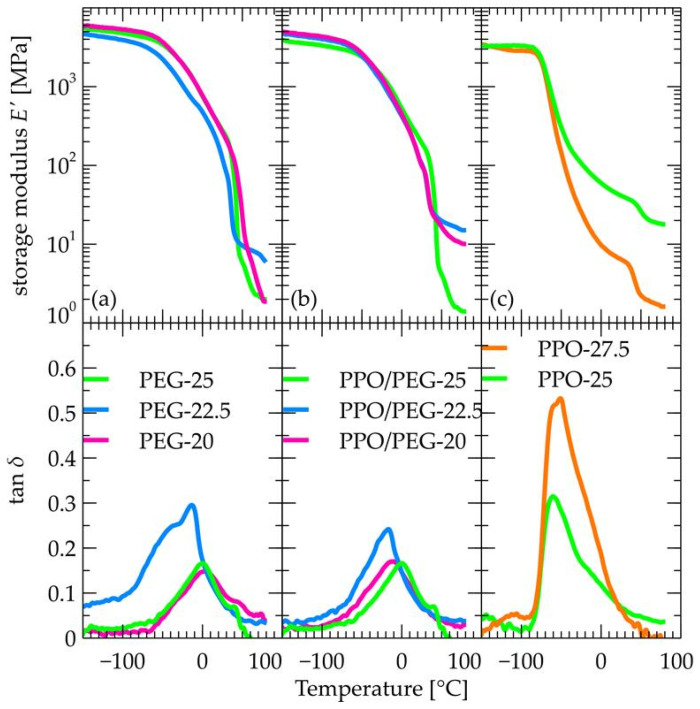
DMA curves of all materials for which a DMA measurement was possible. (**a**) PEG-based materials; (**b**) PPO/PEG-based materials; (**c**) PPO-based materials.

Starting from the PPO-based materials (Figure 7c), the basic feature of the DMA curves is a step in E’ accompanied by a complex peak in tan δ. This is attributed to the α relaxation associated with the dynamic glass transition. Its temperature corresponds well with the calorimetric glass transition temperature, as observed by DSC, and confirms the stability with PPO content observed by DSC.

At higher temperatures, another sharp step occurs around 40 °C. This step is not accompanied by a peak in tanδ, meaning it cannot be attributed to relaxation but rather to a structural change or a phase transition. Interestingly, however, nothing is visible in this region in the DSC experiments. Therefore, we should assume an athermal transition. This is compatible with the effects of transcarbamoylation [75], which is an interesting point to follow in future work. Finally, we would like to note that while composition does not significantly affect the glassy modulus, the rubbery one drops by an order of magnitude when PPO content rises from 25 to 27.5%, indicating that it is the EDC/DADD-rich domains that provide the mechanical integrity of the materials.

For the PEG-containing materials (Figure 7a), the E’ step is much broader and complex, and is accompanied by tan δ peaks, presumably associated with the α relaxation. They occur at much higher temperatures compared to the PPO materials and show a non-monotonic dependence on composition. This should be associated with a slowing down of mobility due to the effects of crystallinity, which is also irregular with composition. Additionally, here, a drop in E’ is observed around 40 °C, which in this case, however, can be easily attributed to the melting of PEG segments.

The glassy modulus of the materials does not show significant variations either with the composition or with polyether type. The values, in the order of GPa, are typical for polyhydroxyurethanes [76] and for conventional polyurethanes [77]. On the other hand, the rubbery modulus depends on both the composition of the materials and the nature of the polyether. This dependence is not monotonic, e.g., in the case of PEG-containing materials, the highest value is shown for the intermediate (22.5 wt%) composition. The intuitive first thought is that the higher the number of rigid segments, the higher the rubbery modulus should be due to reinforcement effects. However, the rubbery modulus is a very complex phenomenon, depending mostly on the degree of microphase separation [61], which, in turn, is very sensitive to the thermal history and composition of the materials [77]. This behavior in NIPUs is an interesting question for future work.

## 3. Materials and Methods

### 3.1. Materials

(2R,3S)-butano-1,2,3,4-tetraol *meso*-erythritol (Wish Pharma, Warsaw, Poland), dimethyl carbonate (DMC), 1,5,7-triazabicyclo [4.4.0]dec-5-ene (TBD), dimethyl sulfoxide (DMSO), and 1,12-diaminododecane (DADD) were purchased from Sigma-Aldrich (Darmstadt, Germany). Poly(ether amines): polyethylene glycol diamine (Mn = 2019 Da) was purchased from Specific Polymers (Castries, France), Jeffamine@ ED2003 (PPO:PEG:PPO = 3:39:3, Mn = 2288 Da), and Jeffamine@ D2000 (PPO, Mn = 2144 Da) was acquired from Huntsman (The Woodlands, TX, USA).

### 3.2. Synthesis of Erythritol Di(Carbonate)

**Scheme 3:**
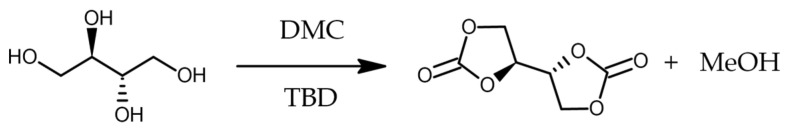
Scheme of the reaction of erythritol to erythritol di(carbonate).

The five-membered di(cyclic carbonate) was synthesized using the transcarbonation reaction (Scheme 3), starting from *meso*-erythritol, according to a modification of the procedure described by Dannecker and Meier [78].

In a 100 mL flask, erythritol (2 g, 16.4 mmol) and TBD (114 mg, 0.820 mmol) were dispersed in DMC and heated to 60 °C in a rotary evaporator until complete dissolution (approx. 40 min) at normal pressure. Then, the temperature was lowered to 40 °C, and the reaction continued at 320 mbar. After 30 min, a white precipitate of erythritol di(carbonate) (EDC) was formed. The product was filtered off after the mixture was cooled down to room temperature and washed with water, yielding a white powder with a melting point of 169 °C, as determined by DSC (lit.: 168−170 °C) [48].

FTIR (ATR): ν = 1806, 1777, 1544, 1472, 1377, 1297, 1203, 1144, 1064, 1028, 977, 897, 773, 744, 715, 533 cm^−1^.

^1^H-NMR (DMSO-d6, 500 MHz): δ (ppm) = 5.16–5.09 (m, 2H, CH), 4.59–4.54 (m, 2H, CH_2_, diastereotopic signals), 4.38–4.35 (m, 2H, CH_2_, diastereotopic signals);

^13^C-NMR (DMSO-d6, 500 MHz): δ (ppm) = 155.7 (CO), 75.6 (CH), 65.8 (–CH_2_–)

### 3.3. General Procedure for Synthesis of Non-Isocyanate Polyurethanes (NIPUs)

The synthesis of materials was analogous to the prepolymer method used for conventional polyurethanes: EDC in DMSO (1 mol/L) was added to a 50 mL round flask and heated to 80 °C to dissolve EDC. Then, the appropriate amine-terminated polyethers were added: PPO, PPO/PEG, or PEG. The amounts of polyethers were pre-calculated so that the polyether to DADD mass fraction had the values shown in Table 1, maintaining a stoichiometry of EDC to amine (polyether and DADD) at a 1:1 molar ratio for all compositions. The reactions were carried out at 80 °C for approx. 48 h, and their progress was monitored by FTIR spectroscopy. The lack of changes in the intensity of the bands originating from the decaying vibrations of the carbonyl group (ca. 1800 cm^−1^) of the cyclic carbonate in favor of the emerging band originating from the vibrations of the carbonyl group of the formed urethane bond (ca. 1720 cm^−1^) marked the end of the first step of the reaction. Then, a pre-calculated (as described above) amount of the low-molecular-weight amine, i.e., 1,12-diaminododecane (DADD), was added to the flask. The reaction continued and was monitored as per the first step until the aforementioned FTIR peaks showed no visible changes (approx. 74 h after the beginning of the process). After the end of this second step, the viscous liquid with a slightly yellow or slightly orange color was poured into a polypropylene mold and placed in an oven at 100 °C for an additional 72 h to complete the reaction between the carbonate and amine groups and evaporate the solvent. A series of NIPUs was obtained based on the variable molar ratio of polyether:DADD, according to the protocol shown in Table 1.

### 3.4. Methods

#### 3.4.1. Fourier Transform Infrared Spectroscopy (FT-IR)

FTIR was applied to track the reaction progress by a Nicolet iS5 spectrometer equipped with a diamond crystal in attenuated total reflectance units, manufactured by Thermo Scientific (Waltham, MA USA). FTIR analysis was conducted in the wavenumber range of 4000–400 cm^−1^, with a scanning resolution of 4 cm^−1^. Deconvolution of the carbonyl band was carried out in the program Grafity (grafitylabs.com) using three terms, of a form proposed by Stancik and Brauns [52], which are the classical Gaussian functions, with an introduced asymmetry:

(3) $$G\left(v\right)=\frac{A}{\gamma \left(\nu \right)}\sqrt{\frac{4\ln2}{\pi }}\exp\left[-4\ln2{\left(\frac{\nu -\nu_{0}}{\gamma \left(\nu \right)}\right)}^{2}\right]$$

with

(4) $$\gamma \left(\nu \right)=\frac{2\gamma_{0}}{1+\exp\left[a(\nu -\nu_{0})\right]}$$

Equation (3) is a Gaussian function with area A, centered at frequency υ. Asymmetry is introduced by substituting its full-width-at-half-maximum parameter with a well-behaved sigmoidal function (4). The asymmetry is expressed by the parameter γ. When = 0, Equation (3) reduces to a simple Gaussian function.

#### 3.4.2. Nuclear Magnetic Resonance (NMR)

NMR spectra were recorded with an FT-NMR 500 MHz spectrometer (JEOL, Tokio, Japan). The measurement temperature was 21 °C with a pulse width of 3.4 µs, 2 s of relaxation, and 1.6 s of acquisition of 3072 scans. DMSO-d6 was used as a solvent, and the spectra shift was referenced to their characteristic shift.

#### 3.4.3. Differential Scanning Calorimetry (DSC)

DSC curves were recorded with a Mettler Toledo 823 calorimeter (Mettler Toledo, Columbus, OH, USA) purged with argon and calibrated with indium. Samples of approximately 8 mg were placed in standard aluminum pans with a pierced lid. The samples were cooled to −80 °C and heated up to 190 °C at 10 K/min to detect morphological changes that would indicate ordering in the as-synthesized materials. Subsequently, a cooling–heating run in the same temperature range and at the same rate was conducted to detect reordering effects.

#### 3.4.4. Wide Angle X-Ray Diffraction (WAXD)

The D2 PHASER apparatus from Bruker (Billerica, MA, USA) was used for the X-ray diffraction investigations. The measurements were performed in the Bragg–Brentanno configuration with the following parameters: the range of the 2*θ* angle was 3–40°, the size of the slit on the lamp was 0.6 mm, and the aperture was 1 mm. The counting time was 0.5°/min with a step of 0.020°. Samples were films of the as-received materials.

#### 3.4.5. Small Angle X-Ray Scattering (SAXS)

The materials containing 25% of the polyether were selected for investigation with small-angle X-ray scattering (SAXS). Curves were recorded with a X-ray setup using a (2D) Hypix 400 X-ray detector (Rigaku, Neu Isenburg, Germany) and a 2.2 kW Cu Long Fine Focus anode lamp. Films of approximately 0.8 mm in thickness were measured in transmission mode, with a windowless holder. The nominal 2*θ* range was −0.005° to 10.0° at a step of 0.005° at a scanning speed of 0.017°/min. The background was measured with an empty holder under the same conditions. The signal of the empty holder at high 2*θ* was considered dark current and was subtracted from all measurements. The corrected signal of the holder was then subtracted from the signal of the sample, taking into account the absorption as follows:

(5) $$I_{s}\left(2\theta \right)=I_{s+h}\left(2\theta \right)-\frac{I_{s+h}\left(0\right)}{I_{h}\left(0\right)}I_{h}\left(2\theta \right)$$

In this Equation (5), *I* denotes intensity, and indices *s* and *h* indicate sample and holder, respectively. Angle was converted to momentum transfer by *q = 4π/λ* sin*θ*, with λ = 1.5406 Å being the wavelength of the source.

#### 3.4.6. Dynamic Mechanical Analysis (DMA)

DMA measurements were conducted with a Netzsch 242C analyzer (Netzsch, Selb, Germany) in tension mode. The specimens had approximate dimensions of 12 × 5 × 0.5 mm. Measurements were run up to 60 °C at 2 K/min, with a frequency of 1 Hz in a nitrogen atmosphere. The reason for the relatively low upper limit is that above those temperatures, the samples became too soft to measure. Some of the materials were not possible to measure, even at low temperatures, either due to poor integrity or due to brittleness. The amplitude of oscillation was set to 20 μm, with a maximum dynamic force of 4 N, and a static to dynamic force ratio of 1:1.

## 4. Conclusions

NIPU elastomers were synthesized via cyclic carbonate aminolysis from erythritol dicarbonate hard segment, PEG-, PPO-, or PPO/PEG-based soft segments, and DADD as a chain extender. After around 74 h, the reaction was completed with a good conversion rate, even without the use of catalysts, due to the high reactivity of the low molar mass cyclic carbonate. Future work could focus on accelerating this reaction by choosing appropriate catalysts.

This formulation resulted in a phase-separated morphology in a fashion very similar to that of conventional polyurethanes (PUs). That is, having a phase-separated morphology in probably two, co-continuous phases: one rich in EDC/DADD (“hard”) segments, and a second one consisting mostly of polyether (“soft”) segments with some hard ones diluted in it. The hard domains are crystalline and also form a periodic structure of approx. 2 nm, likely related to repeat distances between urethane groups. PEG and PPO/PEG retain their crystalline structure within the NIPUs. Although this microphase separation is rather weak when compared to that of conventional polyurethanes, this work shows the potential of NIPUs as a viable alternative.

NMR and FTIR spectra can be interpreted following the paradigm of conventional polyurethanes, showing the expected chemical structure and intersegmental hydrogen bonding. On the basis of the NMR and FTIR results, it is hypothesized that secondary OH from the hydroxy urethane group also takes part in hydrogen bonding in addition to the conventional hydrogen bonding in PUs.

The DSC results reveal that ordering in hard domains is lost during an order–disorder transition around 120 °C, which is significantly lower than that of conventional polyurethanes, presumably due to weaker hydrogen bonding. Reordering is possible during cooling at reasonable rates, indicating that the materials can be used as thermoplastics. The PEO segments in both PEO and PPO/PEG materials are able to crystallize and melt at temperatures close to those of the pristine counterparts. The glass transition temperature of PPO-rich domains is slightly higher than that of pure PPO, indicating the existence of hard segments diluted in this phase.

The materials exhibit good mechanical integrity within a relatively narrow DADD content range. With increasing DADD content, the rubbery modulus of the PPO-based materials increases, which shows that the hard segments, indeed, provide mechanical integrity. However, in the PEO and PPO/PEG materials, it is the crystallinity of the PEG segments that controls the mechanical properties.

In summary, it can be stated that the final properties of the tested NIPUs are the result of a balance between the crystallinity of the soft segments, the order and compatibility of the hard domains, and the ability for microphase separation. Maintaining the crystallinity of PEG and PEG/PPO while modulating the structure of the DADD segments enables the design of materials with diverse morphologies and functionalities. Based on the findings of Beniah et al. (2017) [29], where the use of PTMO soft segments was shown to suppress hydrogen bonding in segmented polyhydroxyurethanes, we propose a complementary strategy described in this work, involving the use of DADD as a relatively long-chain extender. Since these two strategies affect hydrogen bonding via distinct structural mechanisms—soft segment flexibility versus chain extender length and crystallinity—they may act additively or even synergistically when used in combination. This may offer a new avenue for fine-tuning phase separation and related mechanical or thermal properties in NIPU-based materials. The combined effect of those two approaches is feasible because the use of DADD as a universal rigid segment allows for the shaping of the domain order without disturbing the crystalline properties of the soft segments. This work, therefore, provides a solid basis for the further design of NIPUs with controlled structures and predictable functional properties.

In future work, it is worth further studying the kinetics of phase separation, as well as the nature of the phase separation in the meso and molecular scale, by using temperature-resolved spectroscopic techniques.

## Figures and Tables

**Table 1:** NIPU names and compositions.

Sample Name	Polyether	Polyether:DADD [wt%]
EDC-PEG_20	H_2_N-PEG-NH_2_	20:80
EDC-PEG_22.5	H_2_N-PEG-NH_2_	22.5:77.5
EDC-PEG_25	H_2_N-PEG-NH_2_	25:75
EDC-PEG_27.5	H_2_N-PEG-NH_2_	27.5:72.5
EDC-PPO_20	H_2_N-PPO-NH_2_	20:80
EDC-PPO_22.5	H_2_N-PPO-NH_2_	22.5:77.5
EDC-PPO_25	H_2_N-PPO-NH_2_	25:75
EDC-PPO_27.5	H_2_N-PPO-NH_2_	27.5:72.5
EDC-PPO/PEG_20	H_2_N-PPO/PEG-NH_2_	20:80
EDC-PPO/PEG_22.5	H_2_N-PPO/PEG-NH_2_	22.5:77.5
EDC-PPO/PEG_25	H_2_N-PPO/PEG-NH_2_	25:75
EDC-PPO/PEG_27.5	H_2_N-PPO/PEG-NH_2_	27.5:72.5

**Table 2:** Teubner–Strey and Lorentz parameters, as determined by the fit (details in text).

Sample	$d_{TS}$ (nm)	$\xi_{TS}$ (nm)	$d_{L}$ (nm)	$\xi_{L}$ (nm)
EDC-PEG-25	22.41 ± 0.10	3.07 ± 0.03	2.19 ± 0.05	1.46 ± 0.03
EDC-PPO-25	17.50 ± 0.03	3.42 ± 0.01	2.26 ± 0.06	1.68 ± 0.06
EDC-PEG/PPO-25	19.00 ± 0.06	2.86 ± 0.02	2.18 ± 0.04	1.62 ± 0.04

## Data Availability

The original contributions presented in this study are included in the article. Further inquiries can be directed to the corresponding author(s).
